# Correction

**Published:** 1873-01

**Authors:** 


### INDEX.

Abortive treatment of boil, whitlow and felon, pages 48, 370
Abortion, curious case of criminal, 629
Abscess psoas, treatment of, 93
Abscess, preventive of mammary, 368
Abscess of mastoid cells, 630
A case incapsulated pleuritic effusion, by Jno. E. Lochridge, M. D., 65
Acetate of potash and iron, 623
Acne roseaca, 423
Aconite poisoning, 105
Aconite internal use of in neuralgia, 733
Acorn coffee in diarrhœa, 439
Actea, use of in lumbago and rheumatism, 435
Actea racemosa, 491, 665
Acute myringitis, the use of atrophine in, 23
Acute sceloritis, 247
Acute traumatic phlebitis, 249
Acute pneumonia, treatment of, by A. S. Payne, M. D., 385
Acute articular rheumatism, 484
Acnte exanthemata, wet sheets in, 634
Acute rheumatism, baths for, 635
A dangerous paper, 49
Address before the Lowndes County (Miss.) Medical Society by Drs. J. W. M. Shattuck and W. L. Lipscombe. 467, 514
Address delivered before the North Carolina Eastern Medical Convention, held at Goldsboro, N. C., November 4, 1873, by S. S. Satchwell, A M, M D, of New Hanover County, N C, 722
Adhesive plaster fluid, 740
A dichotomous analysis of disease founded on their symptoms, by W T Grant, M D, 325
Affections of the nasal cavities, 233
A good local application in erysipelas, 114
Albumenuria diphtheretic, 686
Alcoholic paraplegia. 172
Alcohol and renal disease, 181
Alterative syrup, 242
Amaurosis from smoking, 58
Amaurosis and ambylopia, treatment by strychnia, 91, 488
Amaurosis, 110
Amaurosis cured by electricity, 117
Ammonia in suspended animation, 616
A mode of operating for radical cure of varicocele, 45
Amputation of cervix uteri by galvanic cautery, 55
Anæsthetics, choice of, 547
Anæsthetic, ether as an, 600
Anœmia, a new treatment for, 629
A new mode of treating puerpural fever, 28
A new means of relieving toothache, 349
A new mode of giving capavia, 312
A new remedy for colic, 479
A new remedy for syphilis, 481
A new treatment for cystitis and irritable bladder of females, 216
Aneurism of arch aorta, 166
Aneurism, abdominal, cured by aortic pressure, 176
Aneurism, thoracic, 556
Anamalculæ in buttermilk, 54
An excel ent pill for dyspepsia, 118
Angina pectoris, electricity in, 48
Ano fistula, operation for, 372
Anodynes and anæsthetics in obstetrics, 739
Anodyne, hops as an external, 495
Anodyne, colloid a new, 424, 435
Antidotes to carbolic acid, 58
Anthrax treated by aspiration, 371
Antiseptic, eucalyptus as an, 493
Anti-hemorrhogic, ergot as an, by T Curtis Smith, M D, 577
Anti-spasmodic, galvanism as an, 663
Antiseptic injections and drainage tubes in empyema, 225
Aperient in eczema, 246
Apoplexy heat, quinia hypodermically in, 49
Apomorphine, as an emetic, 301
Application of electricity, 44
Application to corns, 371
Apparent death, 365
A remedy for colds, 558
A remedy for toothache, 340
Arrest of destruction of lung tissue, 168
Arresting hemorrhage of tonsils, 666
Arnica liniment, 493
Arsenic in paper hangings, 55
Arsenic in cutaneous diseases, 227
Arsenic in constipation, 527
Arsenic in hydrophobia, 559
Arsenic hypodermically for tremor, 627
Arsenic in cancers and tumors, 220
Arsenic, in disease of liver 311, 344
Arsenical paper comp'd, 369
A simple method of arresting epistaxsis, 108
A singular way of opening the bowels, 108
Aspiration and injection of iodine in catarrh of lachrymal sac, 49
Aspiration in hydrocephelus, 50
Asthma, treatment of, 167, 114, 90, 751
Asthma, spasmodic, 620, 367
A substitute for quinia, 225, 104
Atoxia locomotor, 248, 555
Atropia, morphia antidotal to, 112
Atropia, subcutaneously in cholera, 498
Atropia poisoning, 365
Atropized oil in corneal disease, 498
Aural operations in children, 360
Autumnal catarrh, morphia hypodermically in, 485
A useful disinfectant, 50
Balsam of copavia in small-pox and scarlatina, 303
Barnes' dilators, new use of, 744
Baths in acute rheumatism, 639
Baths, small-pox treated by, 685
Baths of warm salt in infantile fevers, 691
Belladonna, action of, 14
Belladonna in ophthalmia, 293
Belladonna in intestinal invagination and hernia, 371, 436
Belladonna to prevent sweating, 639
Beriberi, case of, 425
Beriberis vulgaris in malarial diseases, 687
Bitter taste of quinine disguised, 492
Bisulphide of carbon, 179
Bladder, gelseminem in irritable, 118
Bladder, irritable in females, a new treatment for, 216
Bladder, pain in, 370
Bladder, treatment of, 436
Black cohosh, use of, 539
Black cohosh for rheumatism, etc., 230
Blisters in pneumonia, 46, 689
Blisters, treatment of rheumatism by, 661
Blistering Ointment, Gendreth's, 235
Blood in urine, 52
Bodily quietude in diseases of children, 181
Boils and whitlows, treatment of, 48
Boils to abort, 370
Boils, cause and cure of, 428
Boils, treatment of, 484
Boils, carbolic acid for, 488
Bones, softening of, 57
Borax and nitrate of potash for loss of voice, 692
Bowl, the use of in delivery, 57
Brain diseases, some most important remedies, 96
Bright's disease with dropsy cured by scoparius, 659
Bright's disease, 749
Bromide of calcium, 48
Bromide of potassium in leucorrhœa, 56
Bromide of potassium and atropia in epilepsy, 246
Bromide of potassium in epilepsy, 373
Bromide of potassium, suspension of, 420
Bromide of potassium in cholera, 483
Bromide of potassium in diseases of dentition, 487
Bromide of potassium in Bright's disease, 497
Bromide of potassium, 546
Bromides in summer complaints of children, 243
Bromide of iron, 53
Bromine inhalations of, in diphtheria, 173
Brown, Sequard's neuralgia pill, 177
Burns, warm bath in, 179

Burns, 567, 489
Burns, glycerine of lime in, 743
Caffeine, 173, 690
Calabar bean, tr. of, 58
Calabar bean, 421
Calabar bean for constipation, 103
Calabar bean for hemicrania, 488
Calendula tr., in phagedena, 305
Calomel in diseases of the eye, 172
Camphor in erysipelas, 58
Camphor in ether for erysipelas, 240
Camphor in hospital gangrene, 300, 570
Cancer of the liver, 305
Cancer, 55, 116
Cancer of sublingual glands, 104
Cancer of womb, diagnosis of, 113
Cancer treated by arsenic, 220
Cancer and carbolic acid, 345
Cancer, electrolysis in, 750
Cancrum oris, 43
Cancrum oris, treatment of, 617
Carbolic acid, injurious effects of, 39
Carbolic acid in gonorrhœa, 52, 175
Carbolic acid internally, 170, 228
Carbolic acid internally, 170, 228
Carbolic acid in pruritis, 55
Carbolic acid antidotes, 58
Carbolic acid in burns, 103
Carbolic acid in yellow fever, 106
Carbolic acid in small-pox, 114
Carbolic acid in skin diseases, 180
Carbolic acid in bronchitis, 561
Carbolic acid in cerebro-spinal meningitis, 567
Carbolic acid as a local anæsthetic in boils, 249, 488
Carbolic acid in the treatment of lupus, 615
Carbolic acid, diphtheria treated by, 651
Carbolic acid locally in uterine diseases, 667
Carbolic acid in obstinate leucorrhœa, 309, 433
Carbuncle, nitrate of silver for, 657
Carbazote of ammonia in malarial poisoning, 238
Carbazote of ammonia to supersede the use of quinine, 691
Carcinoma uteri, 356
Carious teeth, 51
Castor oil emulsion, 45
Catarrh, 109, 616, 693
Catarrh of lachrymal sac, 166
Catarrh cut short, 247
Catarrh of nose, 353
Catarrh, autumnal, 485
Catarrh, a prescription for, 498
Catarrh, nasal, chronic, treatment of, 545
Cathartics in albuminuria, 102
Catechu and opium in gleet, 114
Cauterization of urethra, 242
Central galvanism, 676
Cervix uteri, amputation of, 55
Cervical adenitis in children, 84
Cebro-spinal meningitis, 351, 429, 626, 742
Cerebro-spinal meningitis, carbolic acid in, 567
Chancroids, 496
Change of life, 241
Changing direction of wild hairs, 47
Chapped hands and lips, 180
Chalk mixture, 108
Chafing of infants, 634
Chilblains, 39, 303, 494, 499
Children, medicine for, 738
Chloride of potassium, 58
Chloride of zinc, and glycerine in ophthalmia of new-born children, 176
Chloride of ammunium, use of, 619
Chlorate of potass, 18
Chlorate of potass in scrofulous cases, 157
Chlorate of potass in cancerous sores, 428
Chlorate of potass in open carcinomia, locally, 556
Chlorate of potass antiseptic in puerpural cases, 632
Chloral, its administration, 184
Chloral, purification of, 306
Chloral in puerpural convulsions, 422
Chloral, poisonous doses, 736
Chloral, vehicle for, 438
Chloral, local uses of in soft ulcers and ulcerated bnboes, 747
Chloral after chloroform, 442
Chloral in labor, 750
Chloral in tetanus neonatorum, 102
Chloral in sclerosis of middle ear, 490
Chloral and strychnia, 245
Chloral in pertussis, 249
Chloral, depression produced by, 486
Chlorosis, new treatment for, 309, 629
Chloracetic acid for beasts, 178
Chloroform in lead colic, 437
Chloroform in labor, 361
Chloroformization during sleep, 563
Cholagogue, 44
Cholera infantum, tr. iodine in 307
Cholera infantum, treatment of, 566, 622, 633
Cholera, nature and treatment of, 431
Cholera syrup, Reid's 367
Cholera bromide of potass, in 483
Cholera, sulphulic acid a prophylactic in, 492
Cholera, atropia, hypodermically, 498
Cholera morphia, hypodermically, 173
Choice of anæsthetics, 547
Chorea, 245
Churchill's syrup of the hypophosphites, 560
Cimicifuga racemosa in small-pox, 185
Cimicifuga for rheumatism, 230, 491
Cimicifuga in enlargement of spleen, 236
Cimicifuga, 665, 139
Cimicifuga during pregnancy, 539
Citrate of iron and quinine in chlorosis, 309
Citrine ointment for ringworm, 207
Clinical significance of sputa, 285
Clinical report of cases of constitutional syphilis, by T. Curtis Smith, M. D., 403
Clinical history of a case of diphtheritic metritis, etc., by Jno. E. Lochridge, M. D., 595
Coal oil as a deliverative in congestions, 172
Cod liver oil jelly, 560
Cod liver oil etherized, 599
Coffee as a vehicle for quinine, 41
Cold, a remedy for, 558
Colic, a new remedy for, by Jas. T. Alford, M. D., 479
Color of vagina in pregnancy, 207
Colorless tincture of iodine, 219, 439, 499
Colortomy for intestinal obstructions, 232
Collodion styptic, 425
Collodion with sesquichloride of iron, 425
Collodion, mode of employing in erysipelas, 752
Collapse by hypodermic treatment, 485
Conjugal onanizm, 106
Congestive malarial fever, by Benj. Rhett, M. D., 273
Constipation, treatment of, 51, 55
Constipation in children, 491
Constipation relieved by electricity, 182
Constipation, calabar bean for, 103
Control of hemorrhage by ipecac, 117
Convulsions in the new-born child, 437
Conservative surgery in min r operations, 234
Contused wounds, sulphurous acid for, 237
Conjoint use of sulphate cinchona and quinine, 241
Contagiousness of purulent secretions, 244
Consumption, 109, 248, 433
Conjunctivitis granulosa chronica, treatment, 21
Conjunctivitis, chronic, 114, 212
Conjunctivitis, chronic in children, 426
Copaiva, new mode of giving, 312
Corns, cure for, 165, 311, 113, 371
Cornea, treatment of opacities of, 343
Corneal disease, atropized oil in, 498
Cornea, affections, 427
Correspondence by T. S. Heard, M. D., 10
Correspondence, 81, 208, 279
Creasote in anthrax, 180
Creasote, mode of giving, 564
Criminal abortion, 629
Croup, inhalations of bromine in, 107
Croup, treatment of, 108, 469
Croup and diphtheria, 738
Croup, kali brochromium in, 688
Cramps attending pregnancy, 497
Croton chloral, hydrate in painful affections of, 5th nerve, 613
Cundurango not dead, 55
Cundurango, 443
Cutaneous diseases, arsenic in, 227
Cystitis, 110
Cystitis, treatment of, 542
Cysts in the liver in childhood, 172
Danger of performing operations on the cervix uteri, 552
Deodorized tr. iodine, 171

Delirium tremens, 111, 243
Delivering the placenta, 365
Detection of indican in the urine, 431
Determination of life and death of fetus, 442
Diabetes, insipidus, treatment of, 115
Diabetes, melitus, treatment of, 498
Diabetes, 543
Diagnostic sign of cancer of neck of womb, 113
Diagnosis of tuberculosis, 390
Diagnosis, a good lesson, 304
Diarrhœa in children, 31
Diarrhœa, malarial, 40
Diarrhœa of children, wine of pepsin in, 117
Diarrhœa, 249
Diarrhœa, prescription for, 487
Diarrhœa, a mixture for, 561
Diarrhœa, salicin in obstinate, 671
Diarrhœa, chronic, a simple treatment, by A S Payne, M D, 39
Diarrhœa, chronic, iodine in, 371
Diarrhœa, in teething, 736
Dietetic treatment of post-partum, 680
Digitalis poultice for abscess, 225
Digitalis, an anaphdisiac, 436
Digitalis in dropsy, 611
Diphtheria, by W T Taylor, M D, 5
Diphtheria, by T J Heard, M D, 10
Diphtheria, chlorine water for, 12
Diphtheria, Wurm on, 103
Diphtheria, inhalation of bromine in, 107
Diphtheria, temperature in, 562
Diphtheria, treated by carbolic acid, 657
Diphtheria, 735, 741
Diphtheretic albuminuria, 686
Diphtheretic croup, kali cichloratum in, 688
Diphtheroid tonsillitis, 687
Disinfection, 44, 178, 567
Diseases of the Eye, by S M Burnett, M D, 257
Diseases of Children, treatment of, 735
Diseases of children, uses of quinine in, 751
Discolation of the spleen, by A R Kilpatrick, M D., 534
Diseases of ear attributed to quinine, 102
Dropsy, Dr Cameron's domestic remedy for, 732
Dropsy, digitalis in, 611
Dropsy of Bright's disease cured by scoparias, 659
Drainage tubes, how to clean, 734
Dugon oil, 310
Dust in the air passages, 777
Dry earth in surgery, 747
Dyspepsia of liquids, 19
Dyspepsia and derangements of liver, 118
Dyspepsia, new treatment for 626
Dysmenorrhœa, treated by quinia and stramonia, by Dr McIntosh, 683

Earth as a surgical dressing, 741
Eclampsia infantum, by T Curtis Smith, M D, 267
Ectropian, palliative treatment in, 113
Eczema, 182, 430
Eczema, hesitation in curing, 107
Eczema, apperients in, 246
Eczema, rubrum, 681
Editorials, 119, 250, 374, 500, 638, etc.
Effects of color on health, 56
Effects of various liquors on kidney and bladder, 105
Effects of compressed air, 366
Efficacy of ice in gastralgia, 568
Effusion, large pleuretic treated by thirst, 107
Ether as an anæsthetic, 600
Electricity in angina pectoris, 48
Electricity, amaurosis cured by, 117
Electricity in displacements of uterus, 495
Electricity for resuscitation, 551
Electricity, a new method of using, 676
Electro-cautery for piles, 546
Electro-magnetic paper, 482
Elixir of pepsin ond bismuth, 179
Elongation of uvlaa, 440
Empyema, antiseptic treatment, 235
Employment of nux vomica in obstinate vomiting, 247
Endermic application of belladonna for neuralgia, 312
Endometritis, 743
Enemata, nutrative, 234
Enlarged prostate, 653
Epilepsy, 167
Epilepsy, cured by brom. pot. and atropia, 246, 373
Epileptic mania following suspension of bromide of potash, by G D Hodge, M D, 420
Epithlioma cured by creasote, 170
Epistaxis, a simple mode of arresting, 108
Ergotine as a hemostatic, 294
Ergotine in varix, hypodermically, 689
Ergot in treatment of cystitis with hematuria, 296
Ergot, in hemoptysis, 439
Ergot, action of, 499
Ergot, as an anti-hemorrhagic, by T Curtis, M. D, 577
Ergot, use of, 740
Erysipelas, treatment of, 34, 311, 489, 497
Erysipelas, a good local application for, 114
Erysipelas, camphor and ether locally, 240
Erysipelas, mode of employing collodion in, 752
Erythema, 182, 361
Ethiop's mineral in cholera, 437
Etherized cod liver oil, 499
Eucalyptus globulus in intermittent fever. 221
Eucalyptus as an antiseptic, 493
Exanthemata, wet sheets in acute, 634
Exopthalmic goitre, 564
Expectorant mixture, 248
Extraction of foreign bodies from ear, 288
Extracts in powder, 311
External use of fuming nitric acid, 310
Extreme depression and paralysis caused by chloral, 486
External application of verat. veride, 490
Eye, its treatment, 648
Failing heart, digitalis in, 428
Fatal vaccination, by L B Anderson, M D, 205
Febrile diseases in children, use of quinine in, 751
Feeding by the nose. 68
Ferric alum in purpura hemorrhagica, 421
Fever, typhoid, 4, 112, 174, 311, 441
Fever, typhoid, lacto-phosphate of lime in, 174
Fever, typhoid, treatment from hemorrhage of bowels in, 238
Fever, typhoid, baptisa, tinctoria in, 362
Fever, typhoid, treated by strychnia, 364
Fever, typho-malarial, 299
Fever, puerpural, 28
Fever, puerpural, by J G Knox, M D, 591
Fever, yellow, 106
Fever, yellow, by T A Cooke, M D, 194
Fever, scarlet, 42, 628
Fever, treatment of, 35
Fever of Cherokee, Ga, by R Battey, M D, 201
Fever, malarial, by Benj Knett, M D, 273
Fever, rheumatic, cold baths in 441
Fever, by Thos A Cooke, M D, 450
Fever, intermittent, eucalytus in, 221
Fever tree, 238
Fibroid tumors of uterus, 352
Fibrine as a dietectic, 565
Fistula in ano, operation for 372
Fistula in ano, cure for, 492, 544
Fluid ext. of comp'd powder of jalap and senna, 173
Fluid ext. of aconite as a local application, 550
Fluid ext. of chesnut in pertussis, 633
Fluid to restore putrid tissues 591
Formula for podophyllin, 422
Formula for hysteria, 497
Formula for constitutional syphilis, 368
Fœtus, determination of life and death, 442
Freckles, 359
Freckles, ointment for, 533
Functional disease of the eye, by Swann M Burnett, M D, 258
Fungi, vegetable, in the human ear, by A R Kilpatrick, M D, 322
Gallic acid in hemoptysis, 435
Galvanic treatment of bedsores and indolent ulcers, 46
Galvanic cautery to amputate cervix uteri, 55
Galvanism for neuralgia, 170
Galvanism in Grave's disease, 558
Galvanization, central, 676
Galvano-puncture needles, 145
Galvano emesis, 168
Galvano cautery for tracheotomy, 556
Ganglion, treatment of, 53
Gangrene, treatment of, 53
Gangrene, hospital—camphor in, 306, 570
Gastritis, chronic, 53
Gastritis, chronic, nitrate of silver in, 244

Gastric hematemesis, vicarious of regular menstruation produced by malarial causes, by E M Vasser, M D, 536
Gastralgia, chronic, treatment of, 307
Gastralgia, pill for, 312
Gastralgia, ice in, 568
Gelseminum in irritable bladder, 118
Gelseminum in urethral cystis, 179
Gelseminum, value of, 307
Gelseminum gynæcology, 426, 540
Gelseminum, 569
Gelseminum as an antiperiodic, 663
Ginger Wine, 178
Gleet, catechu and opium in, 117
Gleet, formula for, 443, 615
Glycerine lotion, 40
Glycerine lemonade in diabetes, 183
Glycerole of iodine, 555
Goitre, 220, 348, 440, 564
Gonorrhœa, treatment of, 46, 111, 223, 229, 298, 309, 615, 630
Gonorrhœa, carbolic acid in, 52, 175
Gonorrhœa, Reynold's injection for, 47
Gonorrhœa, treated by medicated bougies, 745
Gonorrhœal conjunctivitis, 212
Gout, new theory of, 100
Granville's lotion, a substitute for, 172
Granulated lids, 225
Grave's disease treated by galvanism, 558
Hair-lip, 117
Hair restorative, 610, 556
Hair-dye, Moffat's, 558
Headache, sick, 116, 247, 297, 441
Headache in children, 544
Health, effects of color on, 56
Heart affections, 113
Heart affections, veratria in, 617
Hemorrhage uterine, 50, 367
Hemorrhage of bowels, 238
Hemorrhage of nose, 233, 244
Hemorrhage, controlled by ipecac, 117
Hemorrhage, post par tem, 442, 565
Hemorrhage, uterine, by J. H. Weir, M. D., 279
Hemorrhoidal tumors of the female urethra, 114
Hemorrhoidal suppository, 164
Hemorrhoids, 607
Hemorrhoids, internal, fuming nitric acid for, 631
Hemorrhoids, iodoform in, 542
Hemoptysis, gallic acid in, 435
Hematemesis, gastric vicarious of regular menstruation produced by malarial causes, 536
Hepatic abscess cured by injections of iodized iodide of potassium, 368
Hernial, sac puncture of, 11
Herpes, zoster, treated by electricity, 52
Herpes preputialis, 441
Hiccup, 113
Hooping cough, 184, 226, 291, 426, 564
Hooping cough, carbolic acid in, 593
Hops as an external anodyne, 495
Horse mint, properties of, 245
Household remedies, 624
Hydrocephalus, aspiration in, 50
Hydrocele, 115, 151
Hydrate chloral in labor, 443
Hydrate chloral in selerosis of middle ear, 490
Hydrate chloral in pertussis, 249
Hydrophobia, arsenic in, 559
Hypodermic use of ergot, 175
Hypodermic use of veratrum in meningitis, 351
Hypodermic treatment of collapse, 485
Hypodermic injection of morphia in autumnal catarrh, 485
Hypodermic injection of quinia in heat apoplexy, 490, 693
Hypermetropia, by Swann M. Burnett, M. D., 258
Hysteria, ice to the neck in, 38
Hysteria, formula for, 497
Ice, treatment of tic dolorureaux with, 246
Ice in neuralgia, 499
Icthyosis cured by sulp. of copper, 239
Improved method of taxis, 359
Impotence, 243, 370
Immediate remedy for hemoptysis, 373
Incontinence of urine in old people, tincture of iodine in, 748
Incontinence of urine in children, 50, 310, 364
Indolent ulcers, galvanic treatment of, 46
Induction of premature labor, 369
Infantile paralysis, treatment of, 169
Infantile cholera, treatment of, 566
Infantile fevers, warm salt bath in, 691
Infants, application for chafing in, 308
Infants, management of in hot weather, 475
Inflamed breasts, treatment of, 306, 483
Influence of bromide ammonia on quinia, 366
Influence of belladonna on sweating, 635
Influenza, a remedy for, 553
Ingrowing toe-nail, 185, 298
Inhallation of bromine in diphtheria, 107
Inhallation of bromine, 173
Inhallation poisonous in catarrh, 563
Injurious effects of carbolic acid, 26
Ink spots removed, 50
Insanity in children, 171
Insanity, warm baths in, 179
Intermittent fever, eucalyptus in, 221
Internal use of carbolic acid, 228
Internal use of aconite in neuralgia, 543
Intestinal obstructions, colotony for, 232
Iodine, an air disinfectant, 9
Iodine, deodorized, tincture of, 177
Iodine, tincture in cholera infantum, 307
Iodine, tincture of in dysentery, 307
Iodine and iodide of potash to wounds, 312
Iodine, 364
Iodine in chronic diarrhœa, 371
Iodine in incontinence of urine in old people, 748
Iodine, colorless tincture of, 439, 499, 219
Iodine, tincture of, in vomiting, 731
Iodine, glycerole of, 555
Iodine, tincture of, 568
Iodide of calcium, 111
Iodoform in prurigo and hemorrhoids, 542
Ipecac, in epistaxis, 56
Irritable bladder, gelseminum in, 118
Irritable bladder of females, a new treatment, 216
Irritability of stomach, 237
Irritation of urethra, 443
Iritis, 247
Iron, bromide of, 53
Iron, in rheumatism, 552
Iron, muriate of, in erysipelas, 570
Iron, oxylate of protoxide, 692
Iron, acetate of potash and, 623
Iron, bitter wine of, 746
Is this woman pregnant? by Robert Battey, M D, 417
Itch, 56
Itch ointment, 178
Jalap and senna, fluid ext. comp'd powder, 173
Jaundice, nitrate of silver in, 244
Juniper tar soap, 498
Kali bichlorium in diptheria and croup, 688
Keratitis, small pox, 492
Kidney, effects of various liquors, 105
Knot in the cord, 54
Lacto phosphate of lime in typhoid fever, 174
Lactic acid in dyspepsia, 308
Laryngitis, 416
Laxative powder of jeannel, 499
Lead colic, chloroform in, 437
Lice, to kill, 177, 618
Lime juice and glycerine, 562
Lime, phosphate of, 750
Linimentum am. iodide, 569
Liniment, a good demative, 100
Liniment, Mitchell's, 732
Lips, chapped, 180
Liquor picis alkalinus, 229, 547, 568
Liver, arsenic in, 344
Locomotor ataxia, 248, 555
Lobelia in small doses, 239
Malarial diseases, berberis vulgaris in, 687
Malarial diarrhœa, 40
Malarial fever congestive, 293
Mammary glands, phytolacca for inflamed, 483
Mammary abscess, to prevent, 368
Mammary inflammation, 362
Management of infants in hot weather, 475
Mastoid cells, abscess of, 630
Meat juice, Valentine's, 486
Measles and pneumonia, 183
Measles, carbonate of ammonia in, 557
Medical extracts No. i, ii, by L. B. Anderson, M. D., 341, 528
Medical observations by Fred Horner, M.D., 412
Medicated bougies in gleet, 552
Medicines for children, 738
Meeting of Miss. State Med. Association, 282

Melancholia nitrate of amyl in 631, 744
Memoir of Dr. S. A. Cartwright of N. O., La., formerly of Natchez, Miss., by J. W. M. Shattuck, M. D., 728
Menstruation vicarious, 373
Mercurial trenors, hyoscamine for, 427
Mercury in bronchial asthma, 438
Metro-peritonitis with abdominal abscess successfully opened in the line alba, by A. R. Kilpatrick, 594
Middle ear, dry catarrh of, 490
Mixed vapors for anæsthesia, 182
Monsel's salt for nervi, 494, 632
Morphia, hypodermic use of, 173
Morphia and verat virdi in pericarditis, 174
Mosquito netting as a surgical dressing, 486
Mucilage for office use, 499
Muriate of opium, 171
Muriate of ammonia, 548, 619
Nævi cured by Monsel's salt, 494, 632
Nasal hemorrhage, 233, 244
Nausea of pregnancy, 738
Needles, improved galvano puncture, 145
Neuralgia, 485, 433, 548.
Neuralgia of testicles, 163
Neuralgia and siatica cured by turpentine, 495
Neuralgia, galvanism in, 170
Neuralgia pill, 177
Neuralgia, internal use of aconite in, 733
Nevada soda, 620
Night sweats, 44, 171
Night sweats of phthisis, 430, 487
Nitrate of silver in jaundice and chronic gastritis, 244
Nitrate of silver in testitis and carbuncle, 657
Nitrate of silver in epilepsy and chorea, 302
Nitrate of silver, poisoning by, 248
Nitrate of amyl in spasmodic asthma, 312
Nitrate of amyl as an antispasmodic, 549
Nitrate of potassa and borax for loss of voice, 692
Nitric acid, fuming for internal piles, 631
Nitric acid, for hemorrhoids, 371
Nose, catarrh of, 353
Nose, feeding by, 684
Nurses' sore mouth, 550
Nutrative enemata, 234
Nux vomica in the diarrhœa of exhaustion, 308
Nux vomica in melancholia, 631
Oakum dressing, 43
Obstinate vomiting, nux vomica for, 247
Occlusive dressing, 37
Ointment for itch, 178
Ointment of oxide of zinc, 438
Ointment citrine for ringworm, 207
Ointment blistering, 235
Ointment for freckles. 533
Oil coal as a divivative, 172
Oleum terebinth in acute affections of middle ear, 160
Onychia malignant, nitrate of lead in, 545
Onanism, conjugal, 106
Opacities of cornea, 343
Ophthalmia, strumons in children, 240
Ophthalmia of new-born children, prescription for, 176
Ophthalmia, treated by alcohol, 363
Ophthalmia, belladonna in, 293
Ophthalmitis conjunctivitis, black cohosh in, 230
Ophthalmology, 161
Opium poisoning, rules for giving belladonna for, 496
Opium, muriate of, 171
Opium in wounds of intestines, 184
Orchitis, 242
Otology, 161
Oxide of zinc in night sweats, 434
Oxide of zinc ointment, 438
Ozena, 98
Paper, electro-magnetic, 482
Paper, dangerous, 49
Paper, red, arsenic in, 55
Paralysis, produced by chloral, 486
Paralysis, infantile, treatment of, 169
Paralysis, agitans, treated by hyosciamine, 427
Paraphlegia, 172
Paraphymosis, reduction of, 185
Parturient, morphia as a, 99
Pemphigus, treatment of, 550
Penis, pain in, 370
Pepsin wine in diarrhœa of young children, 117
Pepsin and bismuth, elixir of, 179
Pepsin, 614
Pepsin, its theraputic value, by T Curtis Smith, M D, 641
Pertusis, fl'd extract of castenea verica in, 633
Pertusis, chloral in, 249
Perspiration, excessive of hands and feet, 372
Pessaries, 432
Phimosis, operation for, 634
Phlebitis, acute traumatic, 249
Phosphorus, mixture, 436
Phosphorus pills, 56
Phosphorus in certain diseases of skin, 424
Phosphate of lime, 750
Phtheiriasis, 309
Phthisis, night sweats of, 430, 487
Phthisis, treatment of, 621
Phthisis, temperature of, 360
Phthisical hemoptysis, ergot in, 437
Phytolacca in mastitis, 483, 115, 362
Pills of ferrous oxide, 304
Pill, triplex of Dr Frances, 440
Pill, of phosphorus, 180
Piles, internal, nitric acid for 631
Placenta forceps, use of, 493
Placenta, delivering, 365
Placenta, delivery of by supra-pubic pressure, 731
Plaster, how to remove adhesive, 568
Pleurisy, subacute, 498, 565
Pleuritic effusion treated by thirst, 107
Pneumonia and measles, 183
Pneumonia, blisters in, 46, 687
Pneumonia, acute, 386
Pneumonia, migrans, 541
Podophyllin, formula for, 422, 497
Position in labor, 601
Post-partum dietetic treatment, 680
Post-partum hemorrhage, 442, 565
Potassium, chlorate in catarrh, 435
Premature labor, induction of, 367
Pregnancy, local pain in right side, 364
Pregnancy, black cohosh in, 539
Prophylaxis in scarlatina, 489, 653
Prolapsus uteri, treatment without mechanical means, 287
Propylamine, for rheumatism, 422
Prostrate, enlarged, 653
Pruritus of the meatus, 168
Pruritus vulvæ, 49, 112
Pruritus, obstinate, 110
Pruritus, carbolic acid in, 55
Prurigo and hemorrhoids, iodoform in, 542
Prurigo, 551
Psorasis, 308, 478, 548, 752
Puerpural fever, a new mode of treatment, 28
Puerpural fever, case, 591
Puerpural convulsions, chloral in, 422
Puerpural convulsions, 165, 174
Pulvis glycyrrhiræ compositus ect., 350
Pulse in childhood, 437
Pulse of animals, 623
Pulmonary, emphysema cause, symptoms and treatment, 582
Pulmonary consumption, 248
Purpura hemorrhagica, ferric alum in, 421
Putrid tissues, to restore, 569
Quinine, a substitute for, 104
Quinine, coffee as a vehicle, 41
Quinine, to remove taste of, 43
Quinine and utero gestation, by L. B. Bouchelle, M. D., 480
Quinine by the rectum, 482
Quinine, uses in febrile diseases in children, 751
Recurring tonsillitis, 118
Remarks on the physiological action and therapeutic uses of physostigma venonosum, or the ordeal bean of calabar, by J. Q. A. Hudson, M. D., 705
Remedy for tapeworm, 346, 610
Remedies, household, 624
Resuscitation, electricity for, 551
Revaccination of pregnancy, 236
Rheumatism, iron in, 552
Rheumatism, chronic formula for, 554
Rheumatism, acute varat. veredi in, 561
Rheumatism, treatment of, 484
Rheumatism, propylamine for, 422
Rheumatism, use of actea in, 435
Rheumatism, cold baths in fever of, 441, 635
Rheumatism, 167

Rheumatism, of bladder, 615
Ringworm, citrine ointment for, 207
Rhubarb, sweet tr. of, 553
Salad oil an antidote to strychnia, 752
Salicine in obstinate diarrhœa, 436, 671
Salicine in colliquative diarrhœa, by J. D. Tucker, M. D., 590
Salt in sickness, 746
Salve for lips, 372
Scabies, management of, 249
Scarlatina, prophylaxis in, 481, 675
Scarlatina, 628, 303
Scarlet fever, treatment of, 628, 42
Sceloritis, acute and iritis, 247
Sciatica, treatment of, 357, 166
Scrofulous cases, chlorate potass in, 157
Scurvy, cause of, 177
Senna coffee, 185
Sick headache, 441, 297, 247, 116
Skin diseases, local treatment, 563
Skin diseases, phosphorus in—424
Skin diseases, carbolic acid in—433-180
Sleep, chloroformization during—563
Small-pox keratitis—492
Small-pox treated by tr. iron—432
Small-pox treated by baths—685
Small-pox, to prevent pitting—300
Small-pox, balsam copaiva in—303
Small-pox, cimicifuga in—185
Soap, juniper tar—498
Softening of bones of children, formula for—57
Simple cerate—570
Sore mouth, nurses'—550
Sores, cancerous, chlor. pot. in—428
Speculum, use of in securing placenta—493
Spina-bifida—295
Spleen, dislocation of—534
Sputa, clinical value of—285
Sponge tent in epistaxis—493
Stockings, new use for old—491
Stoke's liniment—498
Stomach, treatment of irritable—237
Stomititis, sulphites in ulcerative—181
Strychnia in amaurosis—488
Strychnia chloral and—245
Strychnia for amaurosis and ambylopia—91
Strychnia in typhoid fever—364
Strychnia poisoning, a case reported by E. T. Henry, M. D.—608
Strychnia in albuminuria—312
Strychnia salid oil, an antidote—752
Stramonium vs. opium poisoning—106
Stumps, dressings of—249
Stuttering relieved—210
Styptic collodion—425
Styptic collodion with sesquichloride of iron—425
Styptic, local—442
Sulphuric acid a prophylactic in cholera—492
Sulphurous acid for psoas abscess—93
Sulphurous acid, uses of—95
Sulphurous acid—632
Sulphurous acid in contused wounds—237
Sulphates of cinchona and quinine—241
Sulphate of cinchona—301
Sulphovinate of soda—302
Sulphites in tonsillitis—224
Summer complaint of children, bromides in—243
Sun strokes—57
Suppository, hemorrhoidal—164
Suppositories for vaginismus—80
Suppuration, sulphate of iron in—305
Surgery, dry earth in—747
Suspended animation, ammonia in—616
Suspended respiration, ice to neck in—38
Sweating, belladonna for—635
Sweet tr. rhubarb—553
Syphilitic skin diseases—554
Syphilitic and skin diseases, local treatment of—563
Syphilitic ulceration of face—41
Syphilis, constitutional in infants—183
Syphilis, stillingia in—185
Syphilis, treatment of—88
Syphilis, formula for—107
Syphilis, stray cases of constitutional—403
Syphilis, Prof. Parker's treatment of tertiary—347
Syphilis, transmission of—349
Syphilis, excellent formula for constitutional—368
Syphilis. subcutaneous injection of mercury in—42
Syphilis, a new remedy for, by Dr. Cull—481
Syphilis, cundurango in tertiary—55-443
Syrup, alterative—242
Syrup, Churchill's hypophosphites—560
Syrup, Reid's cholera—367
Syrups, cupaba—240
Tannin, local uses of, 363, 620
Tape-worm, remedy for—346
Tartrate of iron and potassa in typhoid fever, 4
Tears in children—266
Teething, diarrhœa in—736
Testicle, neuralgia of—163
Tetanus neonatorum, chloral in—102-55
Therapeutical additions—439
Thorocentisis—239
Tobacco, enema—435
Tonsillitis, sulphites in—224-554
Tonsils, enlarged, treatment of—307
Tongue, indication for remedies—632
Tooth, transplanting—49
Tooth-ache, remedy for—340-425
Tooth-ache, a mixture for—443
Trachotomy—301
Trachotomy by the galvano-cautery—566
Tremors, hypordermic injection of arsenic for—627
Trismus nasentium—734
Tuberculosis, diagnosis of—290
Tubes, to bend glass—480
Typhoid fever—4-112-174-311-362-748
Typhoid fever—baptisia tinctoria in—364-441
Typhoid fever—buttermilk in—721
Typhus fever and variola—560
Ulcers, chronic, treatment of—618
Ulcers, varicose—619-559
Ulcers—495
Ulcers, dressing for—742
Urine, injection of healthy in diseased bladder—437
Urine, microscopical examination of—1
Urticaria from privy seats—312
Uterine hemorrhage—45-167-367
Utero gestation—quinine in—480
Vaccination from a revaccinated person—175
Vaccination, revaccination of pregnant women—236
Vaccination, fatal—205
Vaccine virus, how to preserve—51
Vaginismus—80
Vagina, cancer of—211
Varicose veins—112
Varicocele—109
Varicocele, radical cure of—45
Vascular cornea, pains—225
Veratrum vivide—355
Veratrum, antidote to opium—116
Veratrum with morphia in pericarditis—174
Vermifuge syrup—311-497
Vinegar as a protection against small-pox—669
Vitality and its co-ordinate forces—586
Vomiting, nux vomica and salts of strychnia in—247
Vienna paste, the use of to relieve pain—751
Warts—87
Warts, chlorcetic acid in—178
Warts, treatment of—429
Wash for falling hair—549
Whitlow and boils, carbolic acid in—52, 48
Whitlow, treatment of—354
Wild cherry, infusion of—560
Wine, ginger—178
Wounds of intestines, opium in—184

### The Southern Medical Record.

EDITED BY

T. S. POWELL, M.D.,

AND

W. T. GOLDSMITH, M.D.

---

### ASSOCIATE EDITORS:

**GEORGIA.**

Robert Battey, M.D.

R. C. Word, M.D.

W. L. Kirkpatrick, M.D.

James A. Long, M.D.

W. L. M. Harris, M.D.

H. L Battle, M.D.

W. C. Musgrove, M D.

L B. Bouchelle, M.D.

John C. Drake, M.D.

E. F. Knott, M.D.

Thomas Burdell, M.D.

E. H. W. Hunter, M.D.

V. S. Hitt, M.D.

J. E. Pope, M.D.

J. A. Hunnicutt, M.D.

A. H. Brantley, M.D.

J. J. Knott, M.D.

J. C. Wisdom, M.D.

**ALABAMA.**

J. C. Knox, M.D.

Geo. F. Taylor, M.D.

J. B. Barnett, M.D.

S. M. Hogan, M.D.

D. S. Hopping, M.D.

J. T. Searcy, M.D.

J. F. Hill, M.D.

**MISSISSIPPI.**

C. B. Gallaway, M.D.

Edward Lea, M.D.

S. V. D. Hill, M.D.

W. B. Harvey, M.D.

J. W. M. Shattuck, M.D.

E. T. Henry, M.D.

T. P. Lockwood, M.D.

Robert Kells, M.D.

**VIRGINIA.**

Charles S. Lewis, M.D.

John H. Claiborne, M.D.

F. Horner, Jr., M.D.

R. J. Preston, M.D.

A. S. Payne, M.D.

J. E. Lockridge, M.D.

J. S. Whorton, M.D.

Wm. O. Owen, M.D.

Sam'l P. Ripley, M.D.

Benj. Blackford, M.D.

E. A. Drewry, M.D.

Rob B. Tunstall, M.D.

A. J. Terrell, M.D.

W. F. Barr, M.D.

L. B. Anderson, M D.

S. L. Jepson, M.D., W. Va.

J. M. Lazzell, M.D.

**SOUTH CAROLINA.**

E. T. McSwain, M.D.

G. M. Rivers, M.D.

**TENNESSEE.**

J. D. Tucker, M.D.

Swan M. Burnett, M.D.

**TEXAS.**

S. M. Welch, M.D.

T. J. Heard, M.D.

**NORTH CAROLINA.**

Geo. A. Foote, M.D.

Thos. F. Wood, M.D.

H. Kelly, M.D.

J. R. Hughes, M.D.

S. S. Satchwell, M.D.

A. B. Pierce, M.D.

E. A. Anderson, M.D.

H. T. Bahnson, M.D.

Willis Alston, M.D.

### CORRESPONDING EDITORS:

J. M. Toner, M.D., Washington City, D. C.

John H. Weir, M.D., Illinois.

Thomas J Gallaher, M. D, Pennsylvania.

Ely Van DeWarker, M. D., New York.

Robert Robson, M.D., Indiana.

C. C. F. Gay, M.D., New York.

*Surgeon of Buffalo General Hospital.*

W. T. Taylor, M.D , Kentucky.

J. Orne Green, Boston, Massachusets.

B. W. Stone, M.D., Kentucky,

*Assistant Physician Western Lunatic Asylum.*

James Tyson, M.D., Philadelphia, Pa.

M. H. Henry, M.D., New York,

*Surgeon to New York Dispensary, Department of Venerial and Skin Diseases.*

Wm. R. Whitehead, M.D., New York.

Thomas H. Kearney, M.D., Cincinnati, Ohio.

Benjamin Howard, M.D., New York.

Chas Kearns, M.D., Kentucky.

S. C. Busey, M.D., Washington, D. C.

A. W. Hobson, M.D., Arkansas.

A. W. Calhoun, M.D., now of Berlin, Prussia.

T. Curtis Smith, Ohio.

---

☞ *All Business Letters should be addressed to CHARLES WHITEHEAD, Business Manager, Post Office Box 324, Atlanta, Georgia.*

