# Correction

**Published:** 1873-02

**Authors:** 


CORRECTION.—Owing to wrong "copy" having been placed in the hands of the compositor, the names of a few of our associates were omitted in our last number. The mistake was not discovered until too late to correct. Our friends will find their names "all right" in this issue.

